# Comparative Transcriptome Analysis of the Pinewood Nematode *Bursaphelenchus xylophilus* Reveals the Molecular Mechanism Underlying Its Defense Response to Host-Derived α-pinene

**DOI:** 10.3390/ijms20040911

**Published:** 2019-02-20

**Authors:** Yongxia Li, Fanli Meng, Xun Deng, Xuan Wang, Yuqian Feng, Wei Zhang, Long Pan, Xingyao Zhang

**Affiliations:** 1 Laboratory of Forest Pathogen Integrated Biology, Research Institute of Forestry New Technology, Chinese Academy of Forestry, Beijing 100091, China; lyx020419@caf.ac.cn (Y.L.); mfl@caf.ac.cn (F.M.); XunDeng@caf.ac.cn (X.D.); xwang@caf.ac.cn (X.W.); fengyq@caf.ac.cn (Y.F.); zhangwei1@caf.ac.cn (W.Z.); longpan@caf.ac.cn (L.P.); 2 Co-Innovation Center for Sustainable Forestry in Southern China, Nanjing Forestry University, Nanjing 210037, China

**Keywords:** *Bursaphelenchus xylophilus*, α-pinene, physical and molecular responses, interaction

## Abstract

*Bursaphelenchus xylophilus* is fatal to the pine trees around the world. The production of the pine tree secondary metabolite gradually increases in response to a *B. xylophilus* infestation, via a stress reaction mechanism(s). α-pinene is needed to combat the early stages of *B. xylophilus* infection and colonization, and to counter its pathogenesis. Therefore, research is needed to characterize the underlying molecular response(s) of *B. xylophilus* to resist α-pinene. We examined the effects of different concentrations of α-pinene on the mortality and reproduction rate of *B. xylophilus* in vitro. The molecular response by which *B. xylophilus* resists α-pinene was examined via comparative transcriptomics of the nematode. Notably, *B. xylophilus* genes involved in detoxification, transport, and receptor activities were differentially expressed in response to two different concentrations of α-pinene compared with control. Our results contribute to our understanding of the molecular mechanisms by which *B. xylophilus* responds to monoterpenes in general, and the pathogenesis of *B. xylophilus*.

## 1. Introduction

Pine wilt disease (PWD) is caused by the pine wood nematode (PWN), *Bursaphelenchus xylophilus*, [1] and is an extremely destructive disease. PWD has caused major losses of forests in European [2,3,4,5] and Asian [6,7,8] countries, especially in Japan, China, South Korea, Portugal, and Spain. It was first identified in Nanjing, Jiangsu Province, China in 1982 [9]. Over the past 30 years, PWD has expanded to 16 provinces and areas in China. In the winter of 2017, PWD spread to Fushun, Liaoning Province, where the annual average temperature is 5–7 °C. *Pinus* spp. are the main hosts for *B. xylophilus* [10,11,12]. *Monochamus* spp. beetles [13,14] are the major vectors for *B. xylophilus* infection, as they feed on more than one pine tree during the course of their lives and, thereby, introduce the nematodes into multiple trees through the open sores caused by feeding. Once pine trees are infected, they usually die rapidly, as no effective treatment is available to counteract the disease. Despite many studies concerning PWD and PWNs, the pathogenic mechanism of *B. xylophilus* has not been completely elucidated.

As for the pathogenic mechanism(s) of PWD, several hypotheses have been put forward, such as cavitation caused by terpenoid-induced blockage of water transport in xylem tracheids [15,16], phytotoxins produced by PWN-associated bacteria, and cellulose and pectase secreted by PWNs that degrade the cell wall. Cavitation is a typical pathological characteristic of PWD, especially as the disease progresses. Cavities in infected pines may have different physical manifestations, including pine holes [17,18,19]. One hypothesis that explains how cavitation is involved in the death of PWN-infested trees is that the tracheid cavitation induced by volatile terpenes leads to a water deficit.

Terpenoids are a major type of metabolite of conifers, with monoterpenes being the most important defensive volatile substances, which provide resistance to external plant herbivores and associated pathogen invasion [20,21]. α-pinene accounts for a large proportion of monoterpenes produced by conifers [22]. Zhao and colleagues [23] found significant differences in the concentrations of α-pinene, β-pinene, and β-phellandrene in resistant, partially resistant, and sensitive *Pinus massoniana*, respectively, after inoculation with PWNs, and that the concentrations of the three compounds correlated negatively with *P. massoniana* resistance. α-pinene and β-pinene are the main forms of pinene in pine trees, and are found at a ratio of 1:0.1 [24,25,26]. Kuroda and colleagues [15] inoculated *Pinus thunbergii* seedlings with PWNs and analyzed the xylem derivatives produced by the infections. They found that monoterpenes and sesquiterpenes concentrations increased 2 to 4 times in the xylem soon after PWN invasion, and that these compounds permeated the tracheid via the parenchyma cell wall hole, which eventually led to tube cavitation.

A draft genome sequence of *B. xylophilus* was reported in 2011 [27]. Its availability, in conjunction with previous studies on *B. xylophilus*, including a large-scale proteome study [28], should facilitate additional studies on PWD pathogenicity. To date, however, only a few transcriptome-type studies on the resistance mechanism(s) of masson pines against PWN have been reported [29,30]. Xu and colleagues [31] demonstrated transcriptional responses to PWN infection in non-resistant masson pines. However, no transcriptome data for the response(s) by *B. xylophilus* to terpenoid induction has been reported. Therefore, for the study reported herein, we examined the effects of α-pinene on the physical and molecular responses of *B. xylophilus*, to understand the pathogenesis mechanism(s) of this nematode more clearly. We constructed nine cDNA libraries, one that characterized the response(s) of altered transcription of *B. xylophilus* genes at a relative low concentration of α-pinene (AD, 56.33 mg/mL), a second that examined the changes in *B. xylophilus* gene expression in response to a greater concentration of α-pinene (AG, 214.5 mg/mL), and a control transcriptome (no α-pinene added) for comparison [32]. Genes with altered expression caused by α-pinene were identified by comparing transcriptome data, and some of the results for the two α-pinene treatments were further studied with real-time quantitative reverse-transcription PCR (qRT-PCR).

## 2. Results

### 2.1. Toxic Effects by α-Pinene on B. xylophilus Viability and Reproduction

**Figure 1:**
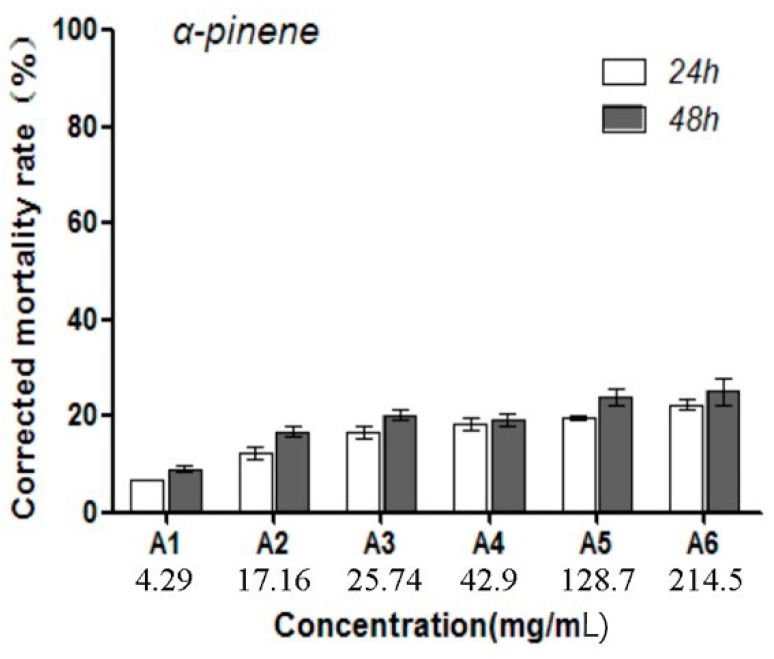
The mortality rates of the pine wood nematodes (PWNs) caused by different α-pinene concentrations. For each treatment, the mortality of PWNs exposed to α-pinene was corrected for the mortality of counterparts exposed to control using aqueous 0.5% Triton X-100 (*m*/*m*) solution. There were three biological replications for each experiment. The error line means standard deviation of the mean.

**Figure 2:**
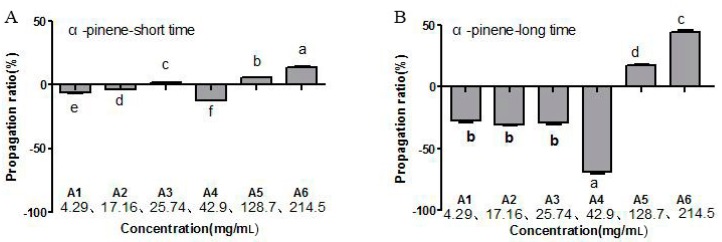
Effect of α-pinene concentration and treatment time on the reproduction rate of PWNs. (**A**) Treatment for a short time. (**B**) Treatment for a long time. There were three biological replications for each experiment. The error line means standard deviation of the mean. Bars with different letters indicate significant differences among the treatments, as defined by Duncan’s test (*p* < 0.05).

**Figure 3:**
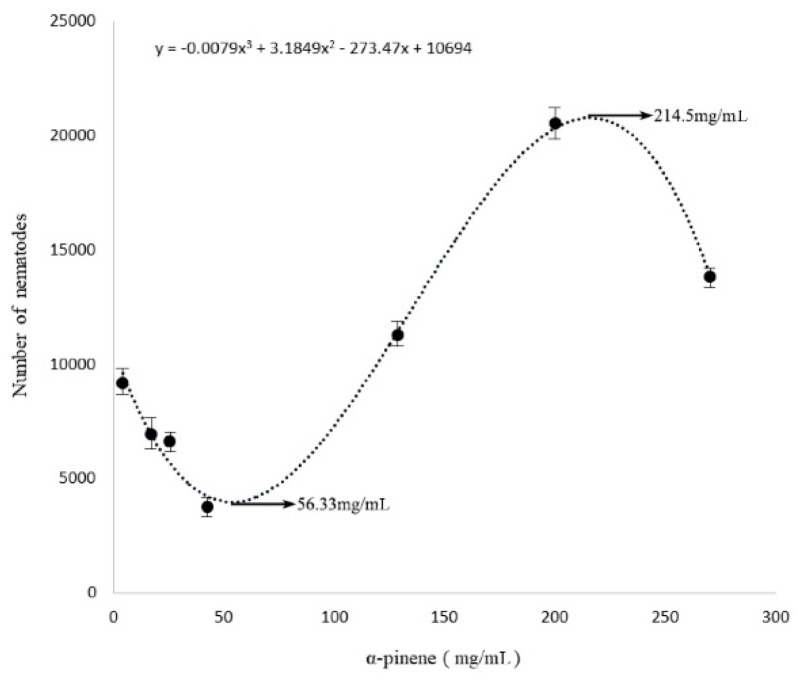
PWN population quantity as affected by α-pinene concentration. The points represent the number of nematodes. The line represents the best fit of the data. There were three biological replications for each experiment. The error line means standard deviation of the mean.

The toxicity of α-pinene on *B. xylophilus* was determined by the cotton ball assay, with the results showing that the *B. xylophilus* mortality rate was significantly different compared with the control (*p* < 0.05), and that mortality increased with increasing concentrations of α-pinene. The highest mortality rate was 22.5% after the PWNs were treated for 48 h (Figure 1). In addition, at 42.9 mg/mL, α-pinene reduced the PWN reproduction rate, whereas the rate increased at greater concentration (128.7 mg/mL; Figure 2). The lowest reproduction rate was observed with 56.33 mg/mL α-pinene (Figure 3).

### 2.2. Transcriptome Sequencing and Data Assembly

The total raw reads were generated from the nematode AD (2,798,113,800 bp), AG (2,267,290,600 bp), and CK (2,121,994,600 bp) transcriptomes (Table 1). The raw sequencing data for the CK, AD, and AG samples were deposited into the NCBI sequence read archive database under the *B. xylophilus* genome sequence (access numbers CADV01000001 to CADV01010432). After removing the adaptor sequences, ambiguous nucleotides, and the low-quality sequences, clean base reads were obtained for the CK, AD, and AG samples, with >85% of the reads in all nine samples exceeding Q20 and Q30 (Table 1), indicating high-quality sequencing. Meanwhile, the number of unique mapped genes was used to evaluating the gene expression. The number of genes that were effectively expressed were 14,796 (CK), 15,159 (AD) and 14,998 (AG), respectively.

### 2.3. Differentially Expressed Genes (DEGs) Found Among the Transcriptomes

**Figure 4:**
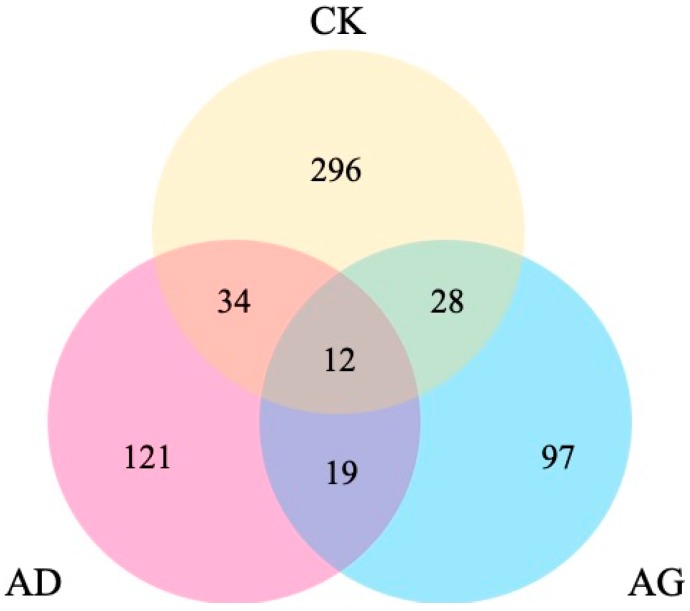
Venn diagram showing the distribution of differentially expressed genes (DEGs) among the transcriptomes. Note: AD, 56.33 mg/mL of α-pinene. AG, 214.5 mg/mL of α-pinene. CK, control.

A comparison of transcriptome profiles among CK, AD, and AG, revealed the DEGs of transcriptomes with each other. The results analysis showed that 121 DEGs were specific to AD and 97 DEGs were specific to AG (Figure 4). For the Gene Ontology (GO) “molecular function” category, 10 upregulated DEGs (CK–AD and CK–AG) were significantly enriched, among which five subcategories were potentially involved in environmental adaption, i.e., oxidoreductase activity, membrane ion channel and transport, steroid hormone receptor activity, hedgehog receptor activity, and acid phosphatase activity (Table 2). In addition, 10 DEGs (CK–AD and CK–AG) were notably downregulated, including those related to ATP binding, cytoskeleton, transmembrane transport, and alcohol dehydrogenase activity (Table 2).

### 2.4. Functional Annotation of the DEGs

Among the upregulated and downregulated genes identified in the transcriptomes, we looked for the major functional PWN genes that might respond to stress induced by α-pinene. These genes included those involved in detoxification, transport, and receptor activities.

#### 2.4.1. DEGs Involved in Detoxification

Compared with the CK transcriptome, four types of genes encoding the proteins CYP450, UGT, SDR, and GST, which are involved in detoxification, were upregulated or downregulated. A total of 20 and 14 CYP450 genes were found to be differentially regulated in the AD and AG samples, respectively, 14 and 15 UGT genes were found to be differentially regulated in the AD and AG samples, respectively, 14 and 17 SDR genes were found to be differentially regulated in the AD and AG samples, respectively, and three and two GST were found to be differentially regulated in the AD and AG samples, respectively. Cytochrome P450s are heme proteins that play central roles in oxidative metabolism and detoxification. Four unigenes (cyp-33c2, cyp-33c4, cyp-33c9, and cyp-32a1) that best matched CYP450 genes were upregulated in the AD and AG samples (Table 3).

#### 2.4.2. DEGs Involved in Transport

A large number of genes encoding channels and transporters were upregulated or downregulated in the PWNs treated with both concentrations of α-pinene, as compared with the control. The number of upregulated and downregulated genes encoding the channels and transporters was greater in the AD sample than in the AG sample. A *B. xylophilus* gene that is similar in sequence to *Caenorhabditis elegans unc-8* was potentially differentially expressed between conditions. In the AG sample, the *unc-8* gene was expressed more than 23.12 times that of the control. The gene *unc-8* is involved in the transport of metabolized toxic substances and ion balance. Conversely, no significant difference was observed for *unc-8* in the AD and CK samples. T09B9.2 is a gene from *C. elegans*, and is a highly expressed member of the transporter superfamily and an important secondary transporter that spans the plasma membrane. A similar sequence was found in *B. xylophilus*, and this similar sequence was upregulated by 2.68-fold and 3.04-fold in the AD and AG samples, respectively. In addition, genes similar in sequence to those of *C. elegans* encoding ATP-binding cassette (ABC) transporters were upregulated 4.55-fold and 2.39-fold in the AD and AG sample compared with the CK sample. Genes sequence similar to those of *C. elegans* encoding other channels and transporters, e.g., TSP-11, TWK-8, T16G1.3, and K09E9.1, were also upregulated. Conversely, other channels and transporters, e.g., F27D9.2, T19D12.9, and Y19D10A.11, were downregulated (Table 3).

#### 2.4.3. DEGs Encoding Receptors

A *B. xylophilus* gene with similar sequence to the *C. elegans* PTR-12, a receptor involved in hedgehog signaling, was upregulated 12.14-fold and 8.45-fold in the AD and AG transcriptomes, respectively. CBG01395 is a gene from *C. elegans*, and a receptor belonging to the nuclear hormone receptor family. A similar sequence is found in *B. xylophilus*, and the expression of this similar sequence was upregulated 11.46-fold and 7.56-fold in the AD and AG transcriptomes compared with the control. Exostosin-2 is a gene from *C. elegans*, and has a characterized function in that organism in soft tissue architecture. A similar sequence is found in *B. xylophilus*, and this similar sequence is differentially regulated—how that tenuous link relates to function in *B. xylophilus* during parasitism is not really clear. The gene encoding exostosin-2 was upregulated 2.91-fold in the AD sample. In addition, the genes that are similar in sequence to those of *C. elegans* encoding the acetylcholine receptor subunit alpha-type deg-3, the cell-growth-regulating nucleolar protein, and the sterol regulatory-element-binding protein 1 were upregulated in the AD sample. Nuclear receptors are primarily responsible for transmitting extracellular signals that regulate the expression of specific genes for control of cell development, stabilization, and metabolism. Genes with similar sequence to those of *C. elegans* encoding nuclear receptors, including shr-86, nhr-19, nhr-3, nhr-40, nhr-62, nhr-70, and nhr-10, were upregulated in the AD sample, and shr-86, nhr-70, nhr-62, and nhr-10 were also upregulated in the AG sample (Table 3).

### 2.5. GO Enrichment Analysis of the DEGs

**Figure 5:**
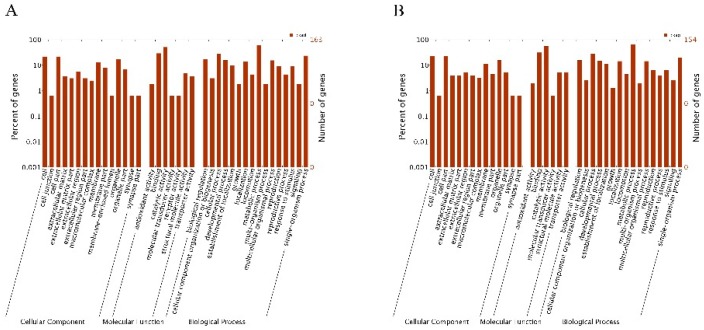
GO assignment of the DEGs in the AD and AG transcriptomes. DEGs in the AD sample (**A**) and in the AG sample (**B**) had GO ID assignments mainly in three categories: cellular component, molecular function, and biological process.

For functional annotation and classification, all DEGs were subjected to GO enrichment analysis. Upregulated genes and downregulated genes were assigned GO IDs and categories (Figure 5). The main GO categories included cellular component, molecular function, and biological process. For the AD sample, GO assignments were as follows. For the biological process category, 18.53% of the DEGs were assigned to metabolic processes (GO: 0008152), and 8.88% were assigned to monomelic metabolic processes (GO: 0044710). For the molecular function category, 54.31% of the DEGs were assigned to catalytic activity (GO: 0003824) and 17.88% were assigned to redox enzyme activity (GO: 0016491). For the cellular component, 15.78% of the DEGs were assigned among the extracellular domain (GO: 0005576) and the endoplasmic reticulum (GO: 0005783) categories. In the AG sample, DEGs were assigned as follows. For the biological process category, 26.21% of the DEGs were assigned to metabolic processes (GO: 0008152) and 11.76% to monomelic metabolic processes (GO: 0044710). For the molecular function category, 56.77% of the DEGs were assigned to catalytic activity (GO: 0003824), and 20.00% to redox enzyme activity (GO: 0016491). For the cellular component category, 13.33% of the DEGs were assigned to extracellular domain (GO: 0005576) or epidermal protein (GO: 0042302).

### 2.6. KEGG Functional Annotation of the DEGs

KEGG functional annotation for the two treatments revealed that 288 and 249 DEGs were enriched in 34 and 44 KEGG pathways, in the AD and AG samples, respectively (Table 4). The major enriched metabolic pathways included those involved in xenobiotic biodegradation and metabolism, carbohydrate metabolism, lipid metabolism, amino acid metabolism, and metabolism of cofactors and vitamins. In addition, transport and catabolism pathways were important for the category cytology process. The endocrine system, digestive, system, and immune system were also significantly enriched in each treatment.

A total of 114 and 112 DEGs were enriched in the top 25 KEGG pathways in the AD and AG samples, respectively, with half of those DEGs involved in xenobiotic biodegradation and metabolism. Genes for CYP450, UGT, and SDR were upregulated in KEGG pathways for metabolism of xenobiotics by cytochrome P450 (ko00980, see Supplementary Figure S1), aminobenzoate degradation (ko00627, see Supplementary Figure S2), drug metabolism—cytochrome P450 (ko00982, see Supplementary Figure S3), and drug metabolism—other enzymes (ko00983, see Supplementary Figure S4).

### 2.7. Verification of Gene Expression by qRT-PCR

**Figure 6:**
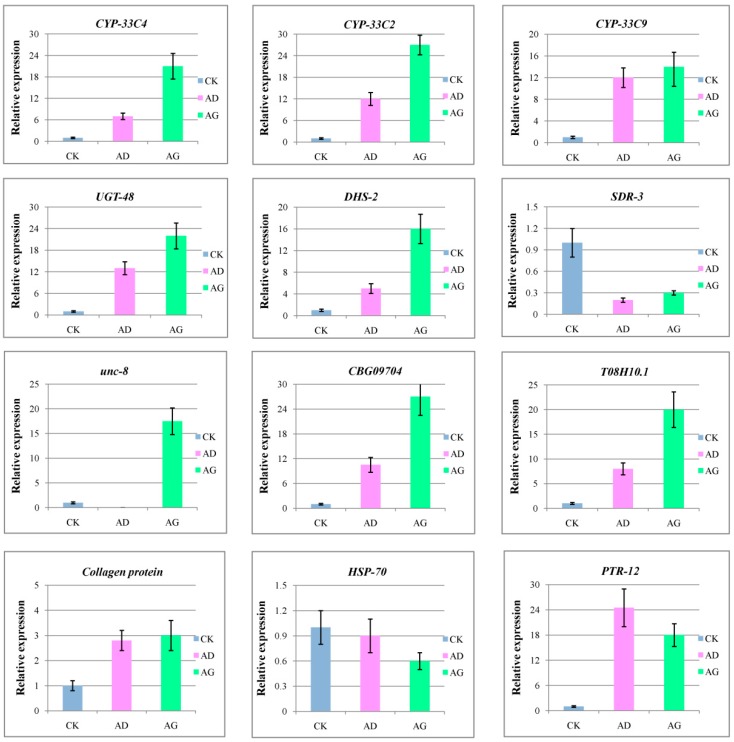
qRT-PCR validation of key candidate DEGs in the AD and AG samples. The CK sample served as the control. Data from the qRT-PCR represent the mean of three replicates, and bars represent standard error.

To confirm the validity of the transcriptome data, we selected a few potentially key DEGs identified in the AD and AG samples that encode detoxification, transport, and receptor proteins for analysis with qRT-PCR. The expression patterns from qRT-PCR were compared with the results of the RNA sequencing expression analysis. The results showed that all the 12 genes had the same expression patterns in the qRT-PCR analysis as in the RNA sequencing analysis, which proved the reliability and accuracy of the RNA sequencing expression analysis (Figure 6).

## 3. Discussion

Terpenes are volatile metabolites that enhance the resistance of plants to biotic stresses, e.g., fungi, bacteria, insects, and nematodes. In this study, the effects of different concentrations of the monoterpene, α-pinene, on the mortality and reproduction rates of the PWN were first assessed to determine the optimal α-pinene concentration that would kill PWNs in vitro. The results of our biological assay indicated that the reproductive rate of *B. xylophilus* was inhibited at a low concentration of α-pinene, but enhanced at a greater concentration. Kong and colleagues [33] identified the main bioactive component of red thyme oil as α-pinene and assessed its effects on the mortality and reproduction rates of PWNs, revealing that the PWN reproduction rate was inhibited, with an LC_50_ for α- and β-pinene of >20 mg/mL. Niu [26] assessed the effects of different concentrations of α- and β-pinene on the PWN and found that each compound inhibited PWN viability at a small concentration and promoted reproduction at a large concentration. The aforementioned results reveal that terpenoids can impact the health of PWNs. Our analysis showed that there was a trough and a peak in the fit of the line to the PWN population quantity as affected by α-pinene concentration (Figure 3). Therefore, it was important that we determine the optimal concentration of α-pinene with which to treat *B. xylophilus* in vitro based on the physiological index, 56.33 mg/mL (AD) and 214.5 mg/mL (AG) of α-pinene, prior to performing our transcriptome experiments.

We constructed two transcriptome databases from total *B. xylophilus* RNA after exposing the nematodes to α-pinene. The expression of certain genes in the α-pinene-treated samples was upregulated, including those encoding oxidoreductase, membrane ion channels and transport proteins, steroid hormone receptors, hedgehog receptors, acid phosphatases, glycosidases, and hydrolases. The expression of certain other genes was downregulated, including those encoding ATP-binding proteins, cytoskeletal proteins, transmembrane transport proteins, alcohol dehydrogenases, histone acetyltransferases, acyl-CoAs, oxidases, α-(1, 3)-fucosyltransferase c, and transferases.

The expression of certain genes was upregulated after the nematodes were exposed to α-pinene, including those of the CYP450, UGT, and SDR families. The PWN is a plant parasite, and its metabolite that is specifically related to detoxification is essential if it is to resist host systemic and induced responses [34]. The PWN completes its parasitic process primarily in the resin of the pine tree. Metabolites found in the pine tree that are toxic to PWNs include terpenoids [35] and aromatic compounds [36]. Genome sequencing analysis of *B. xylophilus* [27] revealed that certain detoxification proteins can reduce the harm caused by host secondary products, and ensure normal feeding and migration so that they can complete their entire life cycle. As previously reported, CYP450s are enriched in the nematode and mediate the degradation of xenobiotics [27,37]. Notably, pinenes are the most abundant monoterpenoids in pine trees. A crude sulfate turpentine from the pine tree was found to be composed of 60–65% α-pinene and 25–35% β-pinene [38]. According to the research of Espada, CYP450 and UGTs were up-regulated at the early stages of infection, and were involved in the detoxification of xenobiotic compounds (α-pinene and β-pinene) [39]. Therefore, to thrive on pine trees, PWNs must have effective mechanisms to degrade pinenes. CYP450 and SDR participate in the phase I detoxification process, and UGT and GST participate in the phase II detoxification process. We found that UGT genes were upregulated and GST and SDR genes were downregulated in the AD and AG samples, suggesting that the CYP450 and UGT activities help PWNs cope with α-pinene stress by detoxifying it. We also used qRT-PCR to amplify three CYP450 genes (CYP-33C9, CYP-33C4, CYP-33C2; Figure 5), the encoded proteins of which are secreted and have an oxidoreductase or transferase activity. Our results will be important for researchers who study detoxification proteins.

Cheng and colleagues [40] showed that PWNs may not be able to completely degrade terpenes, which can only be excreted through redox modification. They also found that bacteria associated with PWNs are needed for complete degradation of terpenes. Detoxification proteins were found to be involved in redox metabolism of terpenes; moreover, channels and transporters appear to be necessary to expel terpenes. For survival, the nematode body must reconcile the difference in external osmotic pressure and respond productively to environmental stress. Unc-8 is a channel protein that maintains the balance between internal and external cellular osmotic pressure and acts as an ion channel in *Caenorhabditis elegans* [41]. Expression of the *unc-8* gene was significantly upregulated in the AG sample, suggesting its participation in detoxification processes. In addition, the genes encoding CBG09704 [42] and T08H10.1 [43] were also upregulated in the AG samples. Given our results, certain PWN genes were upregulated and genes for certain channels were downregulated to ensure efficient detoxification and transport to cope with terpene stress.

In addition to DEGs related to detoxification and transport for which expression was altered, the expression of certain genes encoding receptors was upregulated by α-pinene treatment; the protein products of these genes are involved in normal growth processes or are metabolism-related hormones or stress response proteins. Apparently, in our study, these genes were differentially expressed in PWNs, so that healthy conditions could be maintained during the adverse physiological stress induced by α-pinene. The Hedgehog signaling pathway plays a key role in embryonic development, as it helps regulate cell proliferation and differentiation and coordinates tissue and organ development. This signaling pathway is evolutionarily conserved, as it is needed to regulate vertebrate and invertebrate development [44,45,46]. PTR-12 is a hedgehog receptor with growth regulatory functions and regulates the molting cycle of *C. elegans*, epidermal protein activity, and the growth of multicellular tissues [47]. These functions suggest that this growth regulatory protein in PWNs may respond to the presence of α-pinene. Meanwhile, we selected these potentially key DEGs identified in the AD and AG samples that encode detoxification, transport, and receptor proteins for analysis with qRT-PCR. The expression profiles of the qRT-PCR-amplified genes were very similar to the GO and KEGG functional annotations of the DEGs. According to the GO and KEGG terms found for the DEGs in our study, *B. xylophilus* responds to α-pinene stress by redox-regulated detoxification, with the products being exported via ion or small molecule transport proteins. In addition, metabolic pathways play important roles in the response by PWNs to α-pinene stress. Finally, other genes were upregulated in response to α-pinene, including those for cuticle synthesis and encoding steroid hormone receptors and hedgehog receptors to ensure normal physiological activities of *B. xylophilus*.

Our study should provide a foundation for further verification of gene functions, artificial inoculation, RNA interference, and recombinant expression studies in vitro relevant to the defense mechanisms of PWN.

## 4. Materials and Methods

### 4.1. Materials

The highly virulent *B. xylophilus* strain NXY61 was isolated from wood chips of infested *P. massoniana* in Zhejiang, China and stored at the Laboratory of Forest Pathogen Integrated Biology of Chinese Academy of Forestry, Beijing, China. The *Botrytis cinerea* strain has been maintained in our laboratory.

### 4.2. Toxic Effects of Different α-Pinene Concentrations on the Mortality and Reproduction Rates of B. xylophilus

Treatment was carried out for a short time via the soak method. Six concentrations of α-pinene (4.29, 17.16, 25.74, 42.9, 128.7, and 214.5 mg/mL) [48] were prepared by serial dilution with distilled water containing 0.5% (*w*/*w*) Triton X-100. One concentration of each α-pinene solution and ~500 PWNs (a mixture of juveniles and adults) in 200 µL of water were individually introduced into wells of 96 well plates (Falcon, New York, NY, USA). Control samples contained 0.5% (*w*/*w*) Triton X-100 in distilled water and PWNs. The nematodes were maintained under the same environmental conditions (25 °C for 24 h and 48 h in the dark) as used for colony maintenance [33]. The number of nematodes remaining alive was examined under a microscope 24 h and 48 h after the start of the experiment. Nematodes were considered to have died when their bodies were straight and did not move even after mechanical prodding.

Treatment for long time carried out the cotton ball bioassay [33], which was used to determine whether α-pinene inhibited or stimulated the propagation of *B. xylophilus*. Briefly, suspensions of nematodes in water (~6000 nematodes/mL) were prepared by diluting the standard nematode suspension. Solutions of α-pinene (50 µL each) at different concentrations (4.29, 17.16, 25.74, 42.9, 128.7, and 214.5 mg/mL) were individually injected into 5 mm diameter cotton balls that were separately placed at the center of a mat in 9 cm diameter Petri dishes, and a nematode suspension (60 µL) was then injected into each ball. Controls received an aqueous 0.5% Triton X-100 (*m*/*m*) solution and a nematode suspension. Treated and control nematodes were kept at 25 °C for 7 d in the dark. The nematodes were separated from the culture by the Baermann funnel technique and were counted.

### 4.3. Preparation of PWNs Using Different Concentrations of α-Pinene for Transcriptome Analyses

Given the results discussed in the first section, the 56.33 mg/mL (AD) and 214.5 mg/mL (AG) concentrations of α-pinene in 0.5% (*w*/*w*) Triton X-100 were chosen as representative of a small and large concentration, respectively. Controls received an aqueous 0.5% Triton X-100 (*m*/*m*) solution. PWNs (~3000 nematodes/mL) and one of the two representative concentrations or the control solution was subjected to gentle shaking at 25 °C for 48 h. The samples were then centrifuged for 5 min at 5000× *g* to collect the nematodes for RNA isolation and cDNA synthesis as described in the next section. Each sample was replicated three times.

### 4.4. Total RNA Isolation and cDNA Synthesis

Total RNA from *B. xylophilus* that had been treated with or without α-pinene as described in previous section was extracted with RNAprep Pure Tissue kit reagents (Tiangen Biotech, Beijing, China) according to the manufacturer’s protocol. RNA integrity was confirmed using an Agilent 2100 Bioanalyzer (Mainz, Germany) with a minimum RNA integrity number of 8. The transcriptome samples were prepared using reagents from an Illumina kit (San Diego, CA, USA) following the manufacturer’s instructions. Briefly, poly(A) mRNA was purified from total RNA using oligo (dT) magnetic beads, and then fragmented into short sequences in fragmentation buffer. The cleaved poly(A) RNA fragments were used for first-strand cDNA synthesis with random hexamer primers. The second-strand cDNA synthesis used RNase H and DNA polymerase I. After end repair and adaptor ligation, the products were purified and enriched via PCR to create cDNA libraries.

### 4.5. Sequencing and Assemblage of B. xylophilus Transcriptome DEGs in Response to α-Pinene

Each sample was replicated three times (nine cDNA libraries), and the data of raw reads was averaged over three replications. The nine cDNA libraries were sequenced at the Beijing Genome Institute (Shenzhen, China) on an Illumina HiSeq 2000 platform. Raw Illumina reads were quality-trimmed and filter to remove low quality reads using Trimmomatic v. 0.33 (RWTH Aachen University, Aachen, Germany). Filtered reads were aligned to the reference genome using the transcriptome aligner Tophat v. 2.0.13 (Broad Institute of MIT and Harvard, Cambridge, MA, USA). The Htseq-count function from HTSeq v 0.6.1 (Genome Biology Unit, Heidelberg, Germany) was used to calculate the gene counts. Htseq-count would exclude the mapped reads generated form Tophat with more than one reported alignment. Only the unique mapped reads were used in the downstream analysis. Differential expression analysis of all samples was performed using the DESeq R package (1.10.1) based on the negative binomial distribution [49]. The resulting p-values were adjusted using the Benjamini and Hochberg’s approach, and the p-adjusted value (padj < 0.05) was set as the threshold for significant differential expression [50]. Translated transcript sequences were first searched with Blastx (http://blast.ncbi.nlm.nih.gov/Blast.cgi, accessed on: 20 March 2017) in the non-redundant (nr), Swiss-Prot and KEGG protein databases to retrieve proteins with the greatest sequence similarities, along with their functional annotations. The nr annotations, GO annotations, and functional classifications for the transcripts were characterized using Blast2GO [51,52] and WEGO [53]. Pearson’s correlation coefficient was used to evaluate the correlations with respect to the percentages of transcript representations linked to each GO term and KEGG biological pathway for the three transcriptomes.

### 4.6. Identification of Statistically Enriched GO Terms and KEGG Pathways

The hypergeometric test was used to measure significantly enriched GO terms in the target gene groups in comparison with the control [54,55]. The formula used to measure significance was p= $1-\sum_{i=0}^{m-1}\frac{\left(\begin{array}{c} M\\ i \end{array}\right)\left(\begin{array}{c} N-M\\ n-i \end{array}\right)}{\left(\begin{array}{c} N\\ n \end{array}\right)}$, where N is the number of all genes with a GO annotation, n is the number of DEGs in N, M is the number of genes that are annotated to a certain GO term, and m is the number of DEGs in M. The GO terms with the *p*-value cut-off of 0.005 were deemed to be enriched. In addition, to identify enriched pathways, the hypergeometric test was used in a similar manner to measure the relative coverage of the annotated KEGG orthologous groups of pathways in the background, and pathways with a *p*-value cut-off of 0.005 were considered enriched [56].

### 4.7. Quantitative RT-PCR

Identical portions of each RNA sample were used for qRT-PCR and transcriptome sequencing. The primer pairs (see Supplementary Table S1) for the candidate genes were designed using Primer Premier 6.0. An ABI 7500 Real-Time PCR instrument (Applied Biosystems, Foster City, CA, USA) in conjunction with the SYBR Green detection method were used for qRT-PCR. The cycling parameters were one round at 95 °C for 60 s, and then 40 cycles at 95 °C for 15 s and 60 °C for 35 s. The relative expression of each candidate gene was calculated using the 2^−∆∆Ct^ method [57].

### 4.8. Statistical Analysis

All experiments involving PWNs were independently performed three times, with each treatment replicated five times, and qRT-PCR for each sample was replicated three times. Statistical analyses were performed using SPSS 17.0 (SPSS Inc., Chicago, IL, USA) and Origin 8.0 (OriginLab, Northampton, MA, USA). One-way analysis of variance by Duncan test was used to determine the statistical significance between treatments, with significance being defined as *p* < 0.05.

## 5. Conclusions

We examined the effects of different concentrations of α-pinene on the mortality and reproduction rate of *B. xylophilus* in vitro. The molecular response by which *B. xylophilus* resists α-pinene was examined via comparative transcriptomics of the nematode. Besides, the genes of *B. xylophilus* involved in detoxification, transport, and receptor activities were differentially expressed in response to two different concentrations of α-pinene compared with control. This is beneficial to better understanding the molecular mechanisms of by which *B. xylophilus* responds to monoterpenes in general, and the pathogenesis of *B. xylophilus*.

## Figures and Tables

**Table 1:** Quality of sequencing data for the AD, AG, and CK samples. Note: AD, 56.33 mg/mL of α-pinene. AG, 214.5 mg/mL of α-pinene. CK, control. ExpGene, Expression genes.

Sample	Data (bp)	Reads	Q20 (%)	Q30 (%)	Mapped (%)	ExpGene
CK	2,121,994,600	21,219,946	96.495	89.795	96.77	14,796
AD	2,798,113,800	27,981,138	96.705	90.255	97.06	15,159
AG	2,267,290,600	22,672,906	96.650	90.095	96.94	14,998

**Table 2:** Upregulated and downregulated *Bursaphelenchus xylophilus* genes induced by α-pinene.

Condition	ID	Fold Change	*p*-Value	Annotation
**CK–AD-up**	BUX.s01144.121	29.0980	2.4758 × 10^−13^	CYP-33C4,Oxidoreductase
	BUX.s00351.319	23.1279	0.0364	unc-8,Membrane Ion channel and transport
	BUX.s00713.666	17.9516	5.798 × 10^−12^	F21A3.11,Acid phosphatase activity
	BUX.s00460.317	17.2096	3.57851 × 10^−6^	ugt-48,Glycosyltransferase
	BUX.s01063.115	15.4635	8.46364 × 10^−13^	CYP-33C2,Oxidoreductase
	BUX.s00116.700	14.3489	1.16933 × 10^−11^	CYP-33C9,Oxidoreductase
	BUX.s01337.92	12.1480	0.0176	PTR-12,hedgehog receptor activity
	BUX.s00110.26	11.6515	8.98925 × 10^−6^	CBG09704,oxidoreductase activity
	BUX.s00460.348	11.4609	2.44898 × 10^−8^	CBG01395,steroid hormone receptor activity
	BUX.s00460.315	10.2344	6.54149 × 10^−10^	ugt-48,Glycosyltransferase
**CK–AD-down**	BUX.s01337.103	34.2444	0.016867135	Dimethylaniline monooxygenase
	BUX.s01147.198	33.0521	3.56339 × 10^−16^	Alcohol dehydrogenase 1
	BUX.s00055.304	32.8176	0.020311806	CBR-CUTI-1,Unknown
	BUX.s00116.698	29.7579	7.6292 × 10^−16^	CYP-33E2,Oxidoreductase
	BUX.s00116.725	21.7595	1.37374 × 10^−8^	Oxidoreductase dhs-27
	BUX.s00508.7	21.4028	0.011669587	LMOD1,Cytoskeleton
	BUX.c09083.1	20.3836	1.98965 × 10^−5^	GST-1,Peroxidase and Transferase activity
	BUX.s01147.18	18.8661	8.05309 × 10^−6^	SCAF14537,small GTPase
	BUX.s00609.89	17.8356	0.026861087	Chia-prov protein (Fragment)
	BUX.s01254.326	17.5406	2.43072 × 10^−12^	Elo-6,transferase activity
**CK–AG-up**	BUX.s00116.700	13.2087	3.07567 × 10^−20^	CYP-33C9,Oxidoreductase
	BUX.s01063.115	10.4479	3.01485 × 10^−19^	CYP-33C2,Oxidoreductase
	BUX.s01092.190	8.7500	0.0431	CBN-SRV-15,Unknown
	BUX.s01337.92	8.4583	0.0493	PTR-12,hedgehog receptor activity
	BUX.s01144.121	8.2639	3.21387 × 10^−7^	CYP-33C4,Oxidoreductase
	BUX.s00460.348	7.5662	7.77552 × 10^−9^	CBG01395,steroid hormone receptor activity
	BUX.s00460.317	7.3889	0.0021	ugt-48,Glycosyltransferase
	BUX.s01144.118	7.2917	1.18745 × 10^−6^	CYP-33C1,Oxidoreductase
	BUX.s01092.48	6.8873	6.21794 × 10^−11^	CYP-32A1,Oxidoreductase
	BUX.s01259.89	6.8056	0.0040	GANA-1,Glycosidase and Hydrolase
**CK–AG-down**	BUX.s01337.103	9.1429	0.0475	Dimethylaniline monooxygenase
	BUX.s01268.31	7.1579	8.58 × 10^−5^	Alcohol dehydrogenase 1
	BUX.s00116.725	6.0621	2.60 × 10^−6^	Oxidoreductase dhs-27
	BUX.s01149.11	6.0280	1.65 × 10^−11^	RHY-1,Transferase activity
	BUX.s01281.195	5.6961	1.53 × 10^−9^	Alcohol dehydrogenase 1
	BUX.s01281.74	5.4611	8.24 × 10^−12^	F44E5.4,ATP binding
	BUX.s00560.3	5.4286	0.0034	TTR-28,extracellular space
	BUX.s00364.138	5.3184	1.22 × 10^−10^	F27D9.2,transmembrane transport
	BUX.s00961.158	4.9979	7.15 × 10^−11^	HSP-70,ATP binding
	BUX.s00364.193	4.7950	2.06 × 10^−10^	Acyl-coenzyme A oxidase;

**Table 3:** Summary of DEGs encoding detoxification, transport, and receptor proteins.

Classification	Genes	Function	Fold Change	
			CK–AD	CK–AG
Detoxification	cyp-32a1	Monooxygenase activity	9.4349	6.8873
	cyp-13a8	Monooxygenase activity	0.3504	0.4425
	cyp-33c1	Monooxygenase activity	6.2375	7.2917
	cyp-33c2	Monooxygenase activity	15.4635	10.4479
	cyp-33c4	Monooxygenase activity	29.098	8.2639
	cyp-33c9	Monooxygenase activity	14.3489	13.2087
	cyp-33e2	Monooxygenase activity	0.0336	0.2401
	ugt-47	UDP-glucuronosyl Transferase	4.4247	2.6057
	ugt-48	UDP-glucuronosyl Transferase	17.2096	7.3889
	ugt-49	UDP-glucuronosyl Transferase	3.2175	-
	sdr-1	Dehydrogenase/reductase	5.011	3.325
	sdr-4	Dehydrogenase/reductase	0.1948	0.3949
	dhs-2	Dehydrogenase/reductase	7.528	3.8671
	dhs-27	Dehydrogenase/reductase	4.4334	3.1952
	gst-33	Glutathione S-Transferase	0.1312	0.4045
	gst-39	Glutathione S-Transferase	0.1734	0.4042
Transport	unc-8	Ion channel and transport	23.1279	-
	CBG06849	Integral to membrane	4.9059	5.0114
	CBG15937	Integral to membrane	3.399	3.5515
	T09B9.2	Transmembrane transport	2.6854	3.0404
	TSP-11	Protein; Transmembrane	3.6887	-
	TWK-8	Potassium channel activity	3.9715	2.1194
	ATPase	Sodium/potassium transport	2.3976	-
	ABC	ABC transporter;	4.5513	2.3998
	F44E7.7	Transmembrane transport	-	-
	K09E9.1	Transmembrane transport	-	2.254
	F27D9.2	Transmembrane transport	0.1588	-
	unc-49	Channel activity	2.2265	-
	T19D12.9	Transmembrane transport	0.3451	-
	Y19D10A.11	Transmembrane transport	0.2002	-
Receptor	CBG01395	Steroid hormone receptor activity	11.4609	7.5662
	IFTA-2	Cell growth and regulatory	2.2312	-
	Exostosin-2	Multicellular organismal development	2.9168	-
	PTR-12	Hedgehog receptor activity	12.148	8.4583
	PTR-13	Hedgehog receptor activity	-	2.1063
	SREBP-1c	Sterol regulatory element-binding	4.4802	-
	START	START domain protein; cell division	-	-
	shr-86	Steroid hormone receptor activity	6.7994	3.1469
	nhr-19	Nuclear receptor family member	2.1711	-
	nhr-3	Nuclear receptor family member	2.1206	-
	nhr-40	Nuclear receptor family member	2.706	-
	nhr-62	Nuclear receptor family member	2.4684	2.0637
	nhr-70	Nuclear receptor family member	3.1917	2.5895
	nhr-10	Nuclear receptor family member	2.649	2.9513
	nhr-31	Nuclear receptor family member	-	-

**Table 4:** Top 13 pathways involving DEGs in the AD and AG samples.

KEGG (KO) Term	NO. Genes	
	CK–AD	CK–AG
**Metabolism**		
Carbohydrate metabolism	67	90
Energy metabolism	14	18
Lipid metabolism	77	66
Amino acid metabolism	38	68
Glycan biosynthesis and metabolism	16	17
Metabolism of cofactors and vitamins	42	53
Metabolism of terpenoids and polyketides	3	4
Biosynthesis of other secondary metabolites	11	13
Xenobiotics biodegradation and metabolism	114	112
**Cellular processes**		
Transport and catabolism	57	26
Organismal systems		
Immune system	8	
Digestive system	12	
Endocrine system	14	9

## Supplementary Materials

Supplementary materials can be found at http://www.mdpi.com/1422-0067/20/4/911/s1.

## Abbreviations

PWD	Pine wilt disease
PWN	Pine wood nematode
GO	Gene Ontology
KEGG	Kyoto Encyclopedia of Genes and Genomes
