# Supplementary material for: Comparative Transcriptome Analysis of the Pinewood Nematode Bursaphelenchus xylophilus Reveals the Molecular Mechanism Underlying Its Defense Response to Host-Derived α-pinene
Source: Int J Mol Sci. 2019 Feb 20;20(4):911. doi: 10.3390/ijms20040911 (PMC6412324; doi:10.3390/ijms20040911)
Supplement: Supplementary file 1 [file ijms-20-00911-s001.pdf]

Supplementary Materials

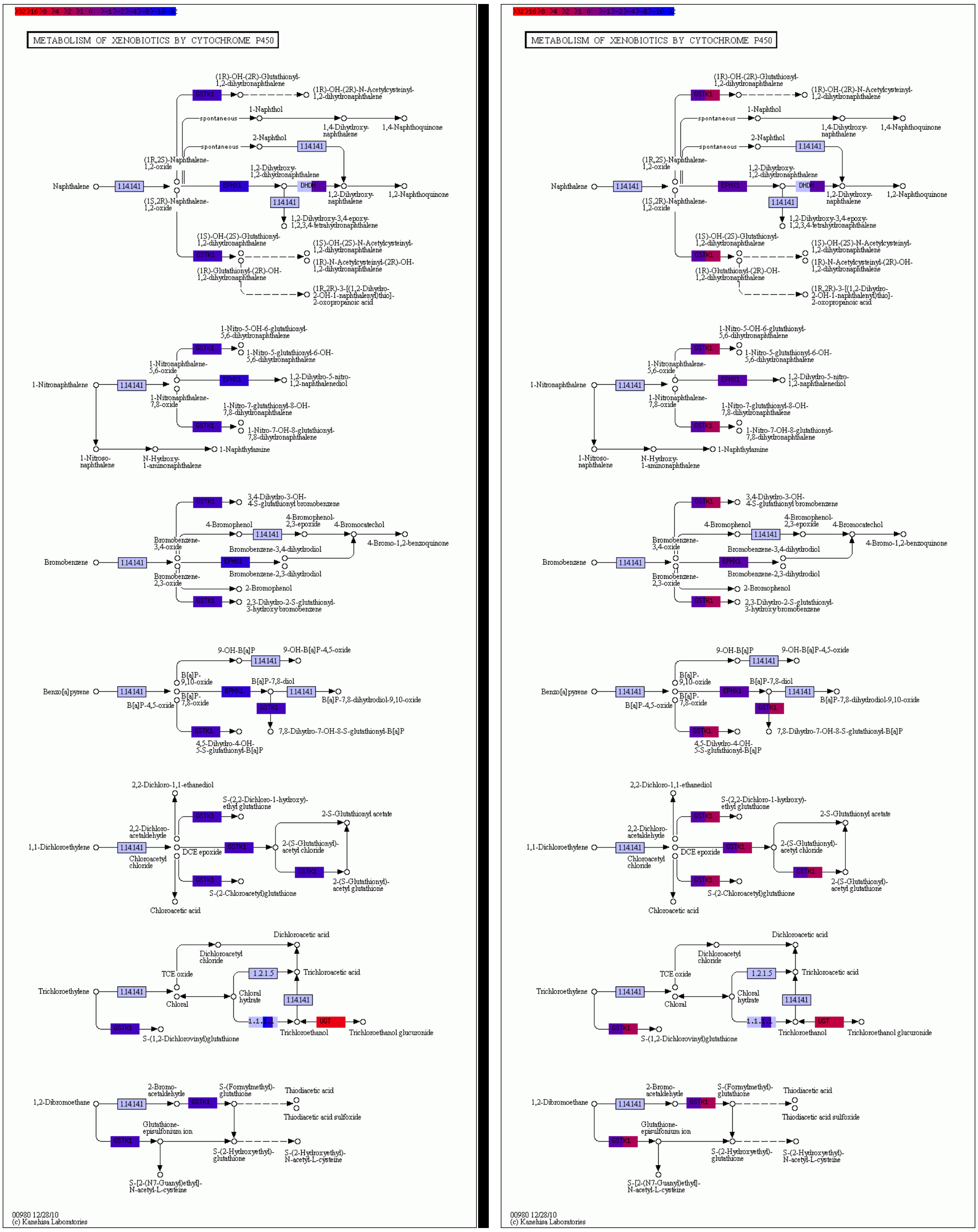

AD **AG**

Figure S1. KEGG pathways for metabolism of xenobiotics by cytochrome P450.

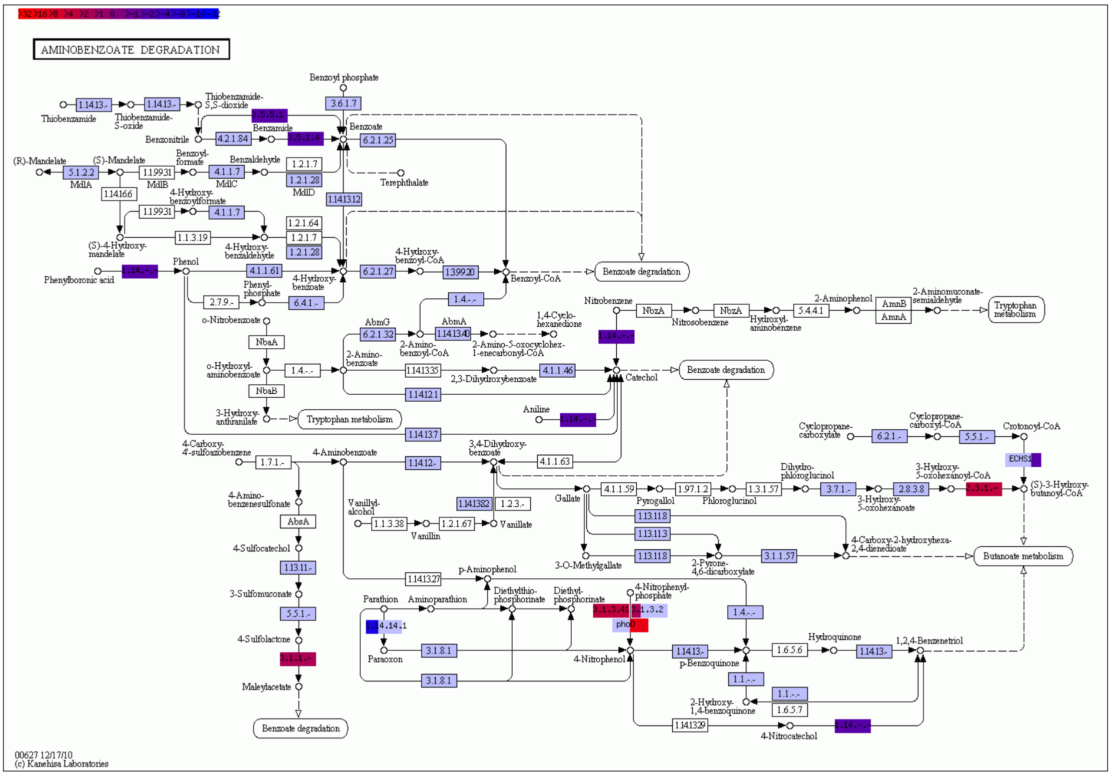

AD

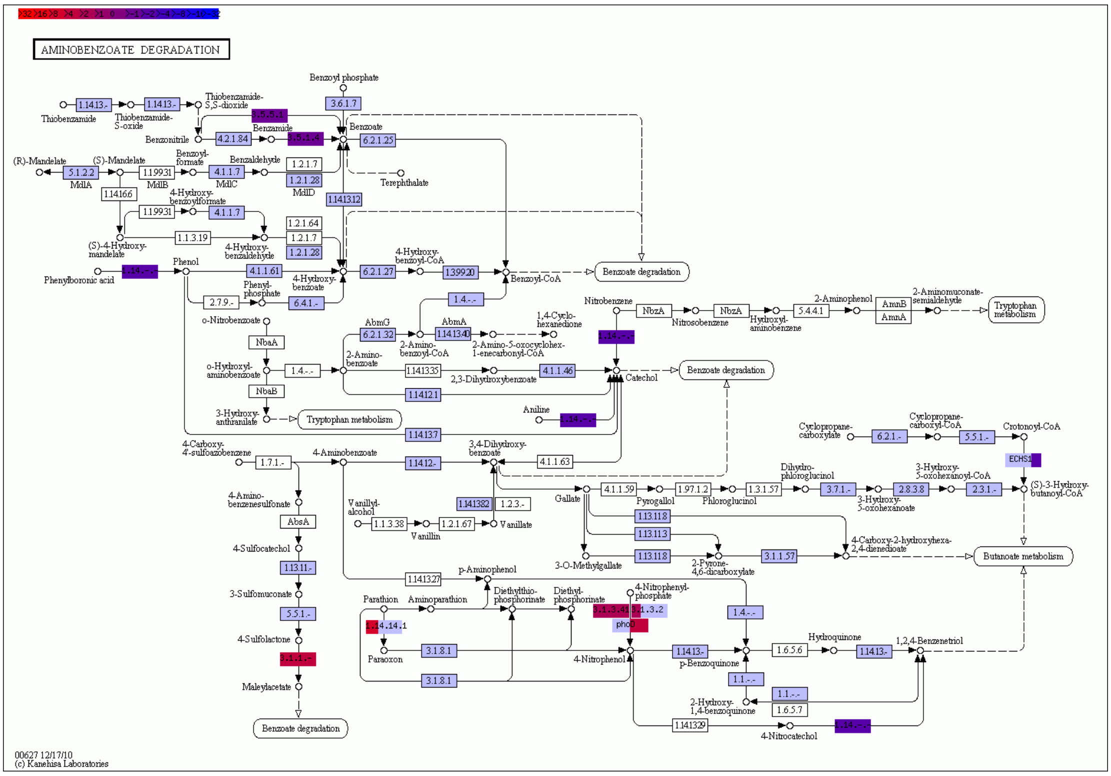

AG

Figure S2. KEGG pathways for aminobenzoate degradation.

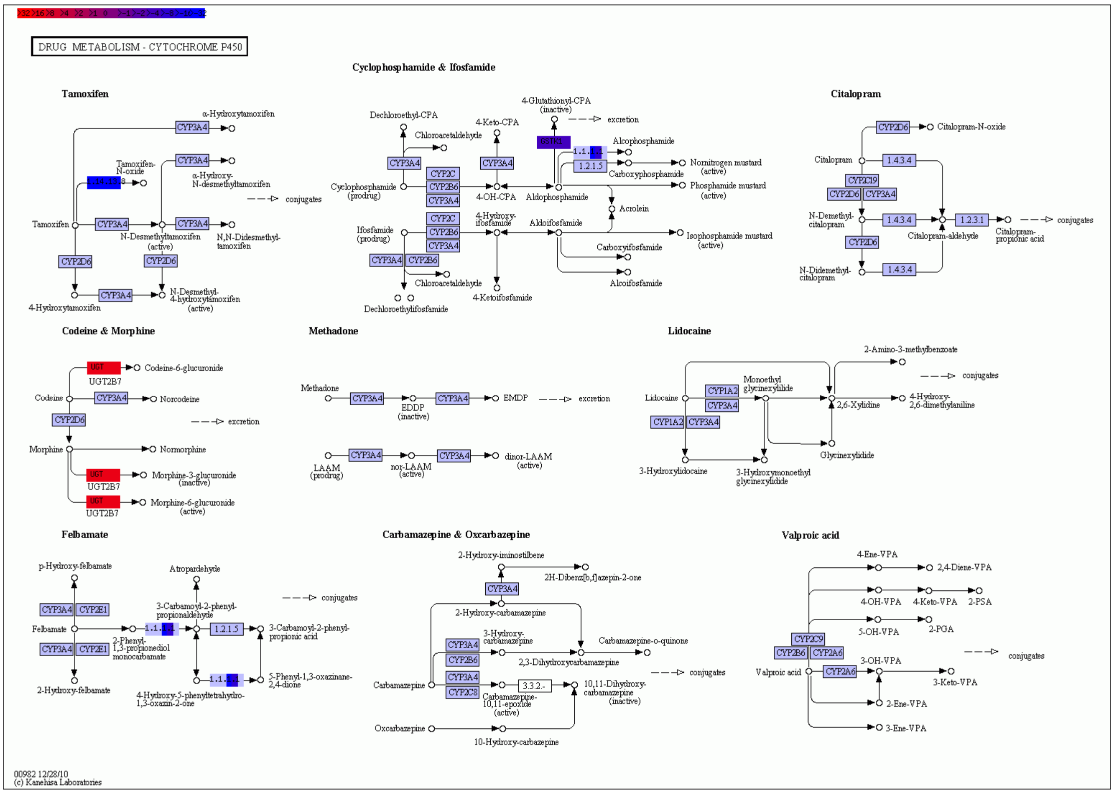

AD

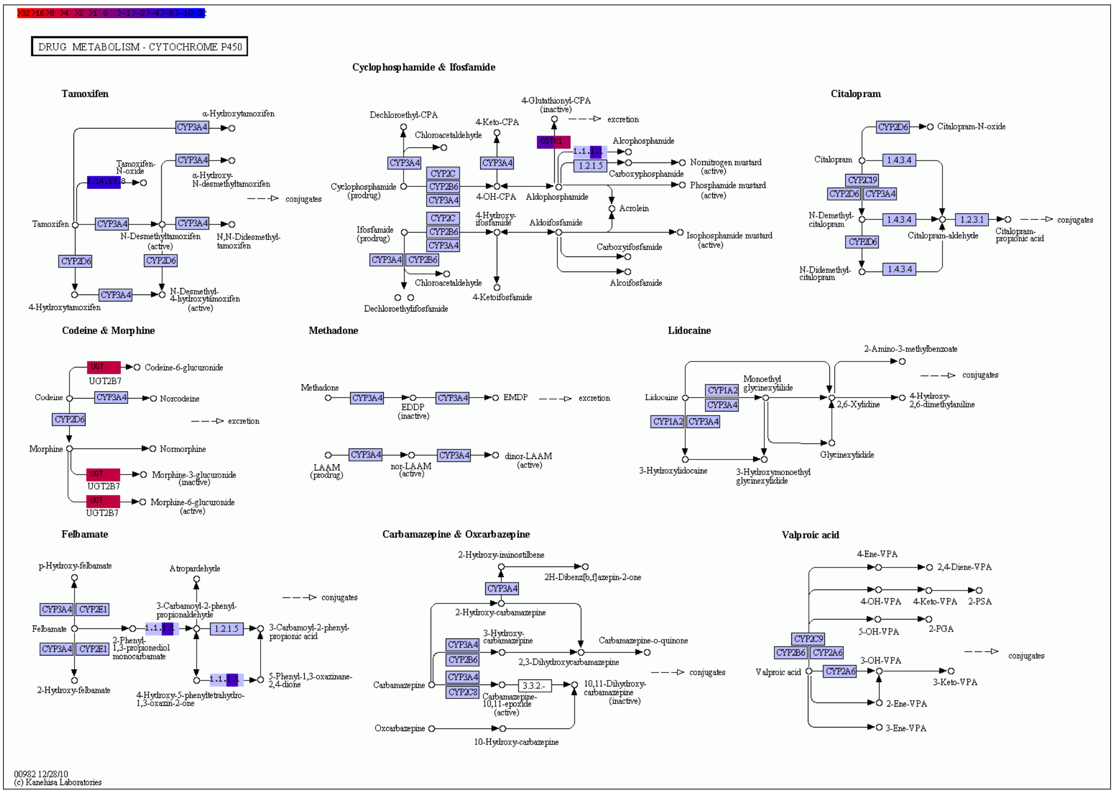

AG

Figure S3. KEGG pathways for drug metabolism-cytochrome P450.

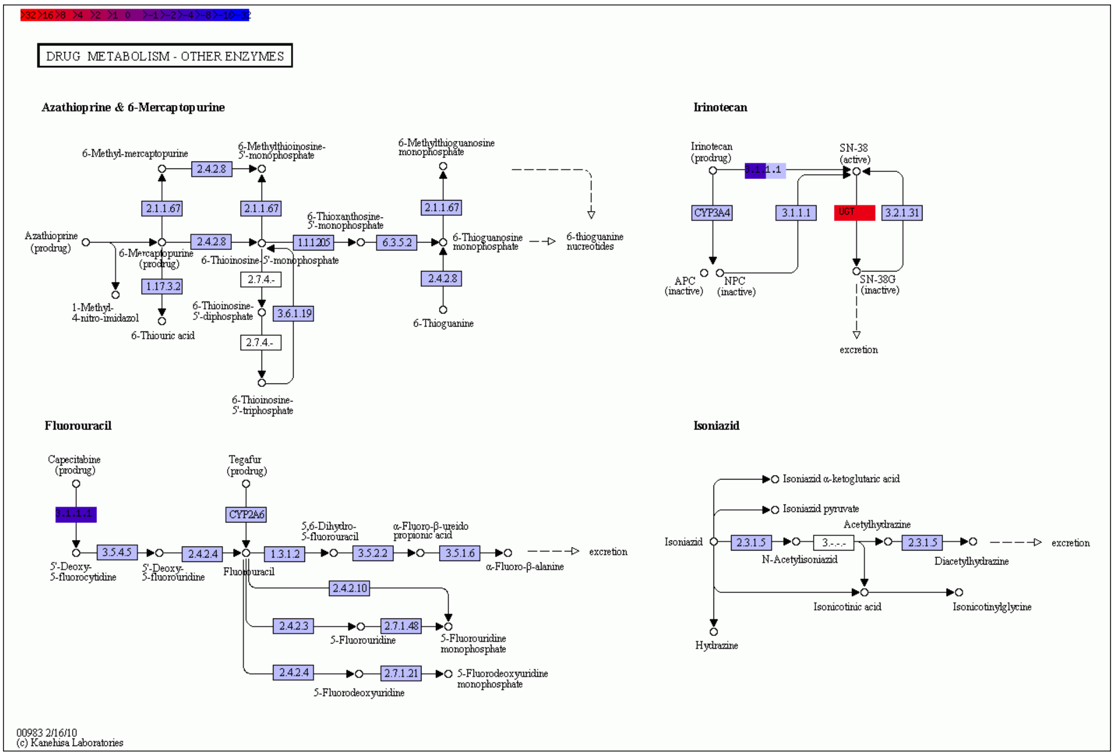

AD

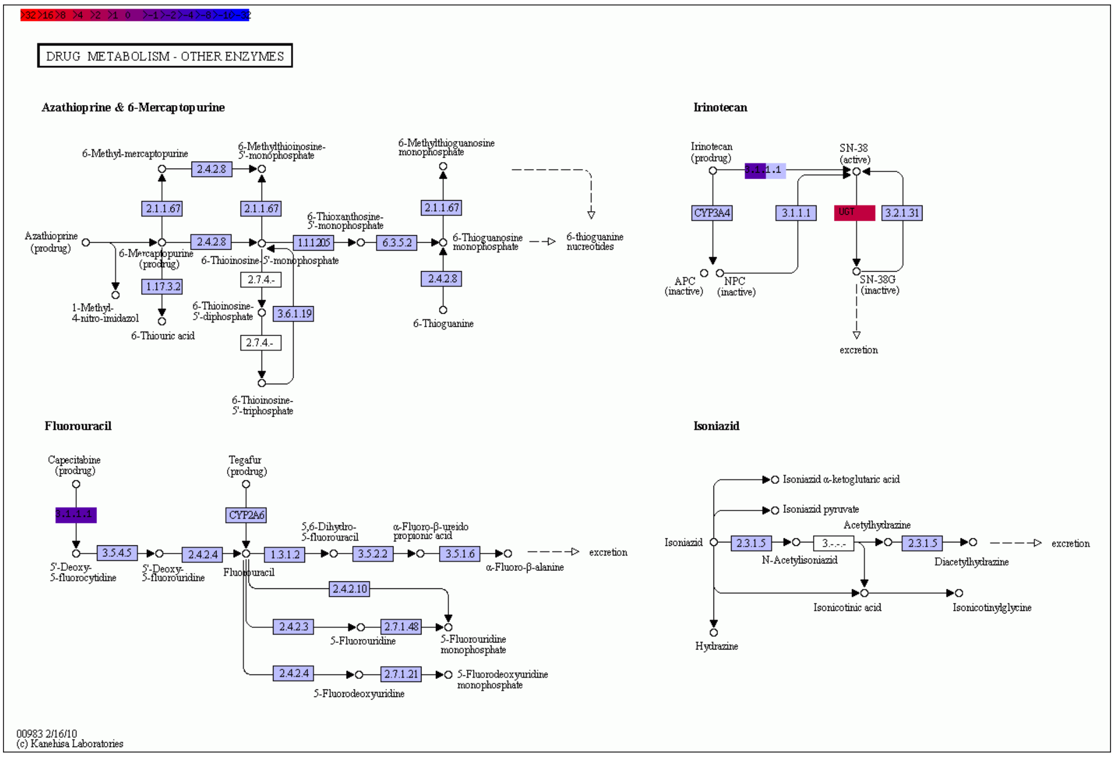

AG

Figure S4. KEGG pathways for drug metabolism-other enzymes.

Table S1 Primer sequence used for qRT-PCR in this study.

Gene	Forward primer	Reverse primer
CYP-33C4	GATACAGATACAGCAAGGAGAAT	AACAACCAGACGGAGGAA
CYP-33C2	CAGTCTGGTTAATGAAGTT	ATAGCCGTTGAGATTGTA
CYP-33C9	CTGATATTGTGGCTTGCTA	AGTCCTACGAGTTGTTGA
UGT-48	GATGAGATGATGGACAATGGT	TTAGTTGAGATTCGCAAGCATA
DHS-2	CGGTATTGCTGCTATTGG	AAGGAGAGGAATGAGAAGAT
SDR-3	TATGAAGGTAATTGAAAGG	ATTATTGACCGAATGAAG
UNC-8	GGAGATGTGGTCAACTTC	CAGAGGCACTAAGATTCG
CBG09704	GTGCTGAAGTTGTTATTC	TTGATGGTCTCGTTAATG
T08H10.1	CCGCTATTATTGTTGTTCCA	TCCCAGAGACCATCAGAT
Collagen protein	GACAGGCTAACAGAACTC	ATTGGATTGCTTCGTGAA
HSP-70	CGAAGGAGAAGTTCAGATG	CTTAGGCGATGAGCAATC
PTR-12	TTATCCAGAGGTCGTTGA	ATTCGCTCGTAGTTCTTG
Reference gene	TCCGTACCCTGAAGTTGGCTAACC	AAGTGGAGACGAGGGAATGGAACC
